# Cytokines and Chemokines at the Crossroads of Neuroinflammation, Neurodegeneration, and Neuropathic Pain

**DOI:** 10.1155/2013/480739

**Published:** 2013-08-12

**Authors:** Geeta Ramesh, Andrew G. MacLean, Mario T. Philipp

**Affiliations:** ^1^Division of Bacteriology and Parasitology, Tulane National Primate Research Center, Tulane University, 18703 Three Rivers Road, Covington, LA 70433, USA; ^2^Division of Comparative Pathology, Tulane National Primate Research Center, Tulane University, 18703 Three Rivers Road, Covington, LA 70433, USA

## Abstract

Cytokines and chemokines are proteins that coordinate the immune response throughout the body. The dysregulation of cytokines and chemokines is a central feature in the development of neuroinflammation, neurodegeneration, and demyelination both in the central and peripheral nervous systems and in conditions of neuropathic pain. Pathological states within the nervous system can lead to activation of microglia. The latter may mediate neuronal and glial cell injury and death through production of proinflammatory factors such as cytokines and chemokines. These then help to mobilize the adaptive immune response. Although inflammation may induce beneficial effects such as pathogen clearance and phagocytosis of apoptotic cells, uncontrolled inflammation can result in detrimental outcomes via the production of neurotoxic factors that exacerbate neurodegenerative pathology. In states of prolonged inflammation, continual activation and recruitment of effector cells can establish a feedback loop that perpetuates inflammation and ultimately results in neuronal injury. A critical balance between repair and proinflammatory factors determines the outcome of a neurodegenerative process. This review will focus on how cytokines and chemokines affect neuroinflammation and disease pathogenesis in bacterial meningitis and brain abscesses, Lyme neuroborreliosis, human immunodeficiency virus encephalitis, and neuropathic pain.

## 1. Introduction

Cytokines are a class of small proteins that act as signaling molecules at picomolar or nanomolar concentrations to regulate inflammation and modulate cellular activities such as growth, survival, and differentiation [1]. Cytokines are an exceptionally large and diverse group of pro- or anti-inflammatory factors that are grouped into families based upon their structural homology or that of their receptors. Chemokines are a group of secreted proteins within the cytokine family whose generic function is to induce cell migration [2, 3]. These “chemotactic cytokines” are involved in leukocyte chemoattraction and trafficking of immune cells to locations throughout the body. Chemokines belong to two categories based on their biological activity, namely, the maintenance of homeostasis and the induction of inflammation [4]. Homeostatic chemokines are involved in immune surveillance and navigation of cells through hematopoiesis and are typically expressed constitutively. Inflammatory chemokines on the other hand are produced during infections or as a response to an inflammatory stimulus and facilitate an immune response by targeting cells of the innate and adaptive immune system. The binding of a cytokine or chemokine ligand to its cognate receptor results in the activation of the receptor, which in turn triggers a cascade of signaling events that regulate various cellular functions such as cell adhesion, phagocytosis, cytokine secretion, cell activation, cell proliferation, cell survival and cell death, apoptosis, angiogenesis, and proliferation [5]. 

**Figure 1:**
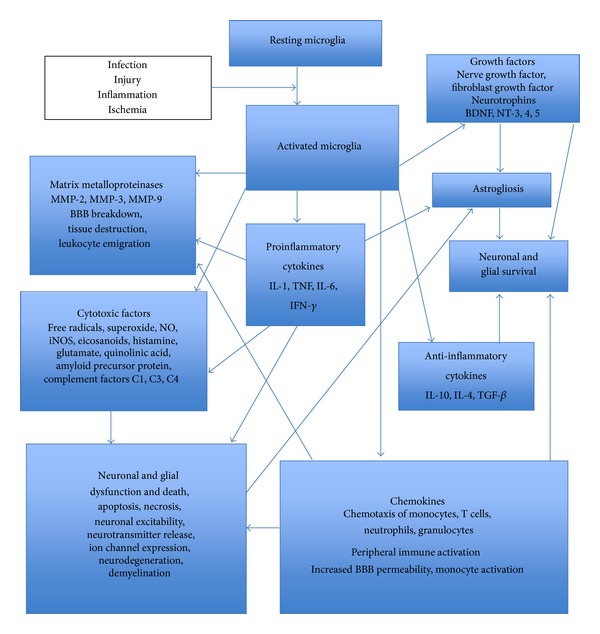
Central role of microglia in neuroinflammation.

In the field of neuroimmunology, the classical view that regarded the central nervous system (CNS) as an immune-privileged site by virtue of its shield, the blood brain barrier (BBB), has evolved to a view of significant CNS-immune system interactions [6]. Cytokines and chemokines are involved in the regulation of CNS-immune system interactions besides being important for the coordination of immune responses throughout the body. They are produced primarily not only by white blood cells or leukocytes but also by a variety of other cells as a response to various stimuli under both pathological and physiological conditions. In the nervous system, cytokines and chemokines function as neuromodulators and regulate neurodevelopment, neuroinflammation, and synaptic transmission. Cytokines and chemokines are crucial to the brain's immune function serving to maintain immune surveillance, facilitate leukocyte traffic, and recruit other inflammatory factors [7]. Upon stimulation by pathogens or abnormal cells, immune cells as well as cells of the nervous system such as microglia (the resident macrophages of the brain), astrocytes, oligodendrocytes, the myelinating cells of the CNS, and Schwann cells in the peripheral nervous system (PNS), endothelial cells of the brain microvasculature, and even neurons can release cytokines and chemokines as well as respond to them by way of cytokine and chemokine receptors [8–10]. Neuroinflammatory processes significantly affect both health and disease of the nervous system by regulating the development, maintenance, and sustenance of brain cells and their connections. In the steady state, microglia protect the nervous system by acting as scavengers of debris and microbial pathogens and by regulating the innate and adaptive immune responses. Pathological states within the nervous system including injury, ischemic stroke [11], and infection [12] can lead to activation of microglia. This in turn can cause release of inflammatory molecules that trigger astrocytes and cells of the immune system to respond to the injury [13]. In the disease state, activated microglia mediate neuronal and glial cell injury and death through production of proinflammatory factors like cytokines and chemokines, glutamate, and reactive oxygen species among others and help mobilize the adaptive immune response and cell chemotaxis, leading to transendothelial migration of immune cells across the BBB and even perpetuation of neuronal damage [14]. The central role of microglia in orchestrating neuroinflammation is described in Figure 1.

In response to injury, neurons produce adhesion molecules and trophic factors that recruit microglial cells and astrocytes. The latter can participate in the ongoing process of damage and repair. In addition to glial cells, the microvasculature also participates in this process. Neurodegeneration is concomitant with astrogliosis, microgliosis, and microvasculature remodeling. Though the trophic factors released initially by astrocytes during astrogliosis aid in tissue repair, these factors amplify the inflammatory response, augment vascular permeability, and result in increased microglial activation and release of more cytokines and chemokines. In states of prolonged inflammation, continual activation and recruitment of effector cells can establish a feedback loop that perpetuates inflammation and ultimately results in neuronal injury [14]. Thus, a critical balance between repair and proinflammatory factors determines the rate of progression and outcome of a neurodegenerative process. 

Understanding the role of proinflammatory cytokines in neurodegenerative diseases is complicated by the cytokines' dual roles in neuroprotection and neurodegeneration. For example, IL-6 has dual roles in brain injury and disease. It is produced during reactive astrogliosis as a response to neuronal damage, acting as a neurotrophin promoting neuronal survival, while elevated levels of IL-6 have also been adversely associated with several brain diseases [15]. 

Some cytokines like IL-1*β* and TNF induce neurotoxicity through elevated glutamate production that results in neuronal excitotoxic death [16]. The inactivation of IL-1*β* and TNF using neutralizing antibodies significantly reduced neuronal death in SK-N-SH cells induced by West Nile Virus [17]. Neuroinflammation and both cytotoxic and vasogenic edema were reduced in IL-1 type 1 receptor-deficient mice conferring neuroprotection in stroke [18]. IL-1*β* also promotes oligodendrocyte death through glutamate excitotoxicity [19]. IL-1*β* and TNF can cause death of oligodendrocytes in a calcium dependent manner [20]. Deletion of the TNF gene ameliorates neurodegeneration in Sandhoff disease (SD), a lysosomal storage disorder [21]. TNF acts as a neurodegenerative cytokine mediating astrogliosis and neuronal cell death in SD, suggesting TNF as a potential therapeutic target to attenuate neuropathogenesis [21]. On the contrary, aggravation of experimental autoimmune neuritis has been observed in TNF-*α* receptor 1 deficient-mice signifying an anti-inflammatory role for TNF in this mouse model [22]. TNF has been implicated in both neuronal death and survival and the level and time of expression determine its final effect on CNS damage or protection [23]. 

Evidence is emerging that chemokines play a role in the physiology of the nervous system, including neuronal migration, cell proliferation, and synaptic activity, besides mediating neuroinflammation. Chemokines are implicated in many diseases of the nervous system. Although their primary role is to induce inflammation through the recruitment of leukocytes by their chemotactic activity, they may also have direct effects on neuronal cells. Chemokines and their receptors are among the key players responsible for communication between neurons and inflammatory cells, and this crosstalk is crucial for normal neurological functioning. Evidence of major roles for chemokines and their receptors in diseases of the brain is accumulating and this system is a potential target for treatment of neurodegenerative diseases [24]. 

Chemokines may induce neuronal death directly through the activation of neuronal chemokine receptors or indirectly through the activation of microglial killing mechanisms. In addition, some chemokines have neuroprotective roles and function as pro- or anti-inflammatory mediators. For example, induction of neuronal MCP-1/CCL2 during mild impairment of oxidative metabolism caused by microglial recruitment/activation exacerbated neurodegeneration in thiamine-deficiency- (TD-) induced neuronal death, while CCL2-knockout (KO) mice were resistant to TD-induced neuronal death, suggesting that the chemokine CCL2 mediates microglial recruitment and neurodegeneration in this model [25]. However, in another system, CCL2 protected neurons from the toxic effects of glutamate and HIV-tat-induced apoptosis [26]. Interestingly, MCP-1-deficient mice showed reduced neuroinflammatory responses and increased peripheral inflammatory responses to peripheral endotoxin insult. These data demonstrate an important role for MCP-1 in regulation of brain inflammation after peripheral endotoxemia [27].

The overexpression of CXCL10 or interferon gamma-induced protein 10 (IP-10) has been observed in several neurodegenerative diseases including multiple sclerosis (MS), Parkinson's disease (PD) HIV-associated dementia, and Alzheimer's disease (AD) [28–31]. CXCL10 elicits apoptosis in fetal neurons by elevating intracellular calcium levels [32]. On the contrary, the signaling of the neuronal chemokine fractalkine (CX3CL-1) and its receptor CX3CR1 has been shown to be neuroprotective, as they reduce the levels of neurotoxic substances like TNF and nitric oxide in activated microglia during neuroinflammation [33]. 

The chemokine IL-8 (CXCL8) that regulates neutrophil migration by signaling through the CXCR2 receptor is markedly elevated by brain injury and is associated with the propagation of secondary damage. Evaluating the function of CXCR2 in posttraumatic inflammation and secondary degeneration by examining Cxcr2-deficient (Cxcr2−/−) mice showed reduced tissue damage and neuronal loss in Cxcr2−/− mice compared to wild-type controls [34]. CXCR1, the receptor for MIP-2 (CXCL2) and CXCR2, has also been implicated in contributing directly to motor-neuron degeneration [35].

The chemokine CXCL1 or GRO1 that is upregulated in brain endothelium in the presence of IL-6 has been identified as a key regulator of granulocyte recruitment into the CNS. Though granulocytes generally exert a protective role in the CNS, they have been shown to be detrimental in experimental autoimmune encephalitis (EAE), the most common model of MS. Administering anti-CXCL1 antibodies attenuated EAE severity suggesting CXCL1 to be a new potential target for the treatment of neuroinflammatory conditions like MS [36].

The chemokine CXCL12 or stromal cell-derived factor 1 (SDF-1) is strongly chemotactic for lymphocytes and it modulates neurotransmission, neurotoxicity, and neuroglial interactions [37]. Growing evidence implicates enhanced expression of CXCL12 and its receptor CXCR4 in the pathogenesis of CNS disorders such as HIV-associated encephalopathy, brain tumor, stroke and MS, making them promising targets for pharmacological intervention [38]. CXCL12/CXCR4 have been shown to promote apoptotic death of dopaminergic neurons in a mouse model of PD [39]. CXCL12/CXCR4 have been suggested to be markers to grade CNS glioblastoma tumor progression. In glioblastoma, a CXCR4 antagonist (AMD3100) showed an inhibition of tumor growth [40]. Thus, several studies using cytokine/chemokine receptor antagonists and deletion mutant mice have provided exciting findings establishing a central role for cytokines and chemokines in mediating neuroinflammation and neurodegeneration and cytokines/chemokines and their receptors represent interesting therapeutic targets in this context.

Immune activation in the nervous system is associated with pathological conditions such as bacterial and viral infections, autoimmune diseases, and inflammatory neurodegenerative disorders including AD, PD, amyotrophic lateral sclerosis, MS [41–44], and Lyme neuroborreliosis (LNB) [45]. Peripheral neuropathies such as Guillain-Barré syndrome [46], PNS Lyme neuroborreliosis [47], demyelinating polyradiculoneuropathies [48], and conditions of neuropathic pain [10, 49] are also accompanied by inflammation. One underlying similarity in all these disease states is the cytokine and chemokine-driven inflammatory response. The dysregulation of cytokines and chemokines is a central feature in the development of neuroinflammation, neurodegeneration and demyelination in the CNS [43, 44], neuritis and axonal degeneration in the PNS [9], and conditions of neuropathic pain [10, 49]. Understanding the involvement of cytokines and chemokines in the pathogenesis of nervous system disorders is relevant for understanding brain pathophysiology and may lead to the development of targeted therapies to treat neurodegenerative diseases. This review will focus on how cytokines and chemokines affect neuroinflammation and disease pathogenesis in bacterial meningitis and brain abscesses, Lyme neuroborreliosis, human immunodeficiency virus encephalitis (HIVE), and neuropathic pain.

## 2. Cytokines and Chemokines in Bacterial Meningitis and Brain Abscesses

Bacterial meningitis is among the top ten causes of death due to infectious agents worldwide. The major meningeal bacterial pathogens are *Streptococcus pneumoniae*, *Neisseria meningitides,* and *Hemophilus influenzae*, although other organisms are capable of causing disease in humans in an age-specific manner [50]. About 50% of patients surviving the infection present with neurological deficits [51]. The pathogen gains access to the bloodstream, penetrates the BBB, and replicates in the subarachnoid space. Immune cells from the peripheral circulation are attracted into the infected subarachnoid space by inflammatory mediators that are produced initially by ependymal cells, meningeal macrophages, and choroid plexus epithelium, followed by local microglia and recently emigrated leukocytes [52–54]. The pathological manifestations of meningitis include increased intracranial pressure, intense brain edema, impairments in cerebrospinal fluid (CSF) flow, seizures, and alterations in cerebral blood flow that may result in focal areas of ischemia and necrosis. During bacterial meningitis, the antibacterial response elicited by the host can be detrimental to neurons and glia in the CNS, due to the toxic effects of cytokines, chemokines, proteolytic enzymes, and oxidants produced locally at the site of infection, in addition to the direct damage caused by pathogens [55].

### 2.1. *Streptococcus pneumoniae* Meningitis

*S. pneumoniae* is the most frequent cause of bacterial meningitis [56, 57]. Effects from meningitis can range from memory deficits, hearing loss, hydrocephalus, cerebral palsy, and seizures. Since the pneumococci can cross the BBB, microglia may respond directly to intact bacteria or to pneumococcal cell wall antigens and produce a wide array of inflammatory mediators including TNF, IL-6, IL-12, keratinocyte-derived chemokine (CXCL1/KC), CCL2/MCP-1,CCL3/MIP-1*α*,CXCL2/MIP-2, and CCL5/RANTES, as well as soluble TNF-*α* receptor II, a TNF antagonist [58, 59]. The production of these inflammatory mediators is associated with the activation of the extracellular signal-regulated protein kinases ERK-1 and ERK-2 via a MAPK intracellular signaling pathway [58, 59].

 The microglial-derived cytokine and chemokine profile represents a double-edged sword since it is effective at eliciting leukocyte recruitment into the CNS for the purpose of antibacterial defenses but at the same time can also contribute to inflammatory mediator-induced neuronal damage by apoptosis triggered, in part, by the inflammatory process via caspase activation [60]. This suggests that strategies to check microglial activation at a point where inflammation is no longer beneficial could reduce damage to surrounding normal parenchyma as a result of bystander destruction. It is suggested that neuronal damage in bacterial meningitis is caused by the dual effects of an overwhelming inflammatory response and the direct effects of bacterial toxins [61]. It is proposed that brain damage in bacterial meningitis leading to long-term neurologic sequelae and death involves several mechanisms. Bacterial invasion and the release of bacterial compounds promote inflammation, invasion of leukocytes, and stimulation of microglia. Leukocytes, macrophages, and microglia release free radicals, proteases, cytokines, and excitatory amino acids, eventually leading to energy failure and cell death. In addition, vasculitis, focal ischemia, and brain edema subsequent to an increase in CSF outflow resistance, breakdown of the BBB, and swelling of necrotic cells cause secondary brain damage.

### 2.2. Cytokines in the Pathogenesis of Pneumococcal Meningitis

The early response cytokines TNF, IL-1, and IL-6 are produced after pneumococcal recognition, which in turn induce upregulation of several adhesion factors on the vascular endothelium, mediating leukocyte influx [62]. An experimental rabbit model of pneumococcal meningitis has shown that the outcome of bacterial meningitis is related to the severity of inflammation in the subarachnoid space and that outcome can be improved by modulation of the inflammatory response [63]. Homologous antibodies to TNF, IL-1*α* and IL-1*β* inhibited leukocytosis and brain edema and moderately decreased BBB permeability in this model of meningitis [64]. A mouse model of *S. pneumoniae* meningitis that mimics several features of human disease describing meningeal inflammation and neuronal damage [65] and an infant mouse model of brain damage in pneumococcal meningitis that exhibits neuronal brain injury in the cortex and hippocampus that reflect the histomorphological findings in the human disease have been established [66]. The infant rat model of pneumococcal meningitis has also been very useful to study the pathogenesis of the disease [67, 68].

The use of KO mice has offered new insights into the role of cytokines involved in the inflammatory cascade during pneumococcal meningitis. Increased mortality and spatial memory deficits in TNF-*α*-deficient mice with experimental pneumococcal meningitis were observed suggesting that TNF plays a role in inflammation and hippocampal injury in bacterial meningitis [69]. Patients with pneumococcal meningitis show increased CSF-TNF-*α*, which correlates with severity of BBB disruption, disease severity, and neurological sequelae [70].

IL-1 is an important proinflammatory cytokine, which is upregulated in brain tissue after the induction of meningitis. Mortality was significantly higher and appeared earlier in the course of the disease in IL-1R (−/−) mice demonstrating that endogenous IL-1 is required for an adequate host defense in pneumococcal meningitis [71]. IL-18 gene-deficient mice showed enhanced defense and reduced inflammation during pneumococcal meningitis suggesting that endogenous IL-18 contributes to a detrimental inflammatory response during pneumococcal meningitis and that elimination of IL-18 may improve the outcome of this disease [72].

The anti-inflammatory cytokine IL-10 has been implicated in playing a role in modulating the immune response by downregulating TNF, IL-6, and keratinocyte-derived chemokine (KC), thereby reducing CSF pleocytosis in pneumococcal meningitis [71]. IL-10 has been shown to repress sepsis-associated hippocampal neuronal damage as a result of pneumococcal sepsis in mice overexpressing IL-10 [73]. Also, intravenously administered recombinant IL-10 reduced the levels of CSF pleocytosis, cerebral edema, and intracranial pressure in a rat model of pneumococcal meningitis [74]. In mice with *S. pneumoniae*-induced meningitis, a deletion of TGF-*β* receptor II on leukocytes is found to enhance recruitment of neutrophils to the site of infection and to promote bacterial clearance. The improved host defense against *S. pneumoniae* was associated with an almost complete prevention of meningitis-induced vasculitis, a major intracranial complication leading to brain damage. The data show that endogenous TGF-*β* suppresses host defense against pneumococcal infection in the CNS [75]. Activin A, a member of the TGF-*β* superfamily and a neuroprotectant that is expressed constitutively in the CSF, has been shown to be upregulated in patients during bacterial meningitis [76, 77]. Cotreatment with activin A and LPS showed increased microglial proliferation and negative regulation of NO, IL-1*β*, IL-6, and TNF in *in vitro*-cultured murine microglia [77].

### 2.3. Chemokines in the Pathogenesis of Pneumococcal Meningitis

Multiple chemokines have been reported to be upregulated in the CSF of patients with pneumococcal meningitis including CCL15, CXCL7, MIF, CCL8, CCL18, CCL20, CXCL5, CXCL-1, CXCL-8, CCL2, CCL3, and CCL4 [78–81]. In animal models of pneumococcal meningitis, additional chemokines have been identified by protein arrays for brain tissues, including CCL9, CXCL-2, XCL-1, CCL-1, CCL11, CCL12, CCL24, CCL25, CXCL4, CXCL10, CXCL12, CXCL13, and CXCL13 [82].

IL-8 (CXCL-8 was found to be chemotactic for neutrophils in the CSF of patients with bacterial meningitis [79]. IL-8 appears to regulate CSF pleocytosis in pneumococcal meningitis from the systemic compartment, similar to that seen for TNF, IL-10, and TGF-*β* [83]. Both MIP-1 (CCL3) and MIP-2 (CCL3) are produced by immune cells resident in the brain and attract monocytes and neutrophils from the bloodstream into the CSF in acute bacterial meningitis [84]. *In vitro*, antibodies against CCL2, CCL3, and CCL4 inhibited monocyte chemotactic properties of CSF from patients with pneumococcal meningitis [79]. The intracisternal inoculation of recombinant CCL3 and CCL4 induced BBB disruption, CSF leukocytosis, and cerebral edema in a rabbit model of pneumococcal meningitis [64]. Of the CXCL chemokines, ENA-78 (CXCL5) was found to be upregulated in patients with bacterial meningitis and exhibited neutrophil chemotactic properties together with IL-8 [80]. A study that evaluated the global response of the BBB to *S. pneumoniae* infection and the specific role of neuraminidase A (NanA), a pneumococcal protein described to promote CNS trophism, revealed that NanA was necessary and sufficient to activate host chemokine induction including IL-8, CXCL-1, and CXCL-2 [85]. In summary multiple chemokines have been reported to be upregulated in pneumococcal meningitis that primarily have a role in attracting leukocytes to the CSF, though the roles of many chemokines in the pathogenesis of the disease have not yet been investigated [86].

### 2.4. *Staphylococcus aureus* and Brain Abscesses

Abscesses in brain parenchyma develop as a consequence of local spread of pyogenic bacteria from the paranasal sinuses, middle ear, or oral cavity, via hematogenous dissemination from a systemic infection or by directly penetrating trauma to the head [87–92]. The most common etiologic agent of brain abscesses in humans is *S. aureus*, besides Streptococci [88]. These infections are characterized by extensive edema and tissue necrosis [89]. The activation of resident microglia is a hallmark of infection [90], in addition to the sequential progression to necrosis during brain abscess evolution. Microglial and astrocyte activation is evident immediately following the entry of bacteria into the CNS parenchyma and persists throughout abscess development in a mouse model [91]. The ensuing abscess formed at the site of infection may result in inflammation accompanied by edema, neuronal toxicity, seizures, and long-term cognitive loss [92]. This murine model has demonstrated that *S. aureus* not only induces brain abscesses but also elicits rapid and sustained expression of numerous proinflammatory cytokines and chemokines including IL-1*β*, TNF, IL-12 p40, CXCL2, CCL2, CCL3, and CCL4 [93–95]. Leukocyte recruitment elicited by microglia into the infected CNS facilitates bacterial clearance during abscess development. Microglia also exert *S. aureus* bactericidal activity. The organism is a potent inducer of numerous inflammatory molecules in microglia such as TNF, IL-1*β*, and CXCL1, among others [96, 97]. The necrotic damage associated with brain abscesses and other CNS infections is accompanied by release of endogenous host molecules that could potentially exacerbate parenchymal necrosis in addition to that mediated by unchecked microglial activation.

Knowledge of the staging of brain abscess in humans is based on findings of CT and MRI scans [98]. During the last decade, an experimental brain abscess model in rats and mice has been established by direct intracerebral injection of live *S. aureus*, [90, 92, 99, 100]. Rodent models mimic accurately the natural course of brain abscess development in humans and have been investigated intensely to understand the mechanisms in disease pathogenesis and the possible treatment modalities.

Brain abscess is typified by a sequential series of pathological changes that have been elucidated in experimental rodent models [90, 92, 99–101]. Briefly, the early stage cerebritis occurs from day 1 to day 3 and is marked by neutrophil accumulation, tissue necrosis, and edema. Astrocyte and microglial activation are seen at this stage and persist throughout abscess development, accompanied by the induction of proinflammatory cytokines and chemokines [90, 94]. From day 4 to day 9 (intermediate or late cerebritis), predominant macrophage and lymphocyte infiltrates are commonly seen. The last or capsule stage is seen from day 10 onwards and is characterized by the formation of a well-vascularized abscess wall that in turn helps to sequester the lesion and protects the surrounding brain parenchyma from additional damage.

However, the immune response that is essential for abscess formation also destroys surrounding normal brain tissue. There is a prolonged expression of IL-1*β*, TNF, and macrophage inflammatory protein-2 (MIP-2/CXCL2), concomitant with a chronic disruption of the BBB in mice with *S. aureus*-induced brain abscess. These changes correlated with the continued presence of infiltrating neutrophils and macrophages/microglia. These observations suggest that the excessive tissue damage that often results from brain abscess may be mediated, in part, by the perpetuation of antibacterial immune responses that are not down-regulated in a timely manner [91]. Similarly, human brain abscess lesions have been found to encompass a large area of the brain, often spreading well beyond the initial focus of infection [102].

On the other hand, cytokines like IL-1*β*, TNF, and IL-6 may exert beneficial effects on the establishment of host antibacterial immune responses. A study that examined the relative importance of IL-1*β*, TNF, and IL-6 in experimental brain abscess using cytokine KO mice showed that IL-1 and TNF play a major role in directing the ensuing antibacterial response, as bacterial burdens were significantly higher in both IL-1 and TNF-*α*-KO mice compared to wild-type mice which correlated with enhanced mortality rates in KO mice [91].

Neutrophils that are the major peripheral cell infiltrate associated with brain abscesses are the source of proinflammatory cytokines such as TNF that serve to amplify the antibacterial immune response [103]. Neutrophils can also exert bactericidal activity through the production of reactive oxygen intermediates and nitrogen intermediates and hydrolytic enzymes that can directly destroy bacteria; however, the continuous release of cytokines and bactericidal products by neutrophils can also contribute to tissue damage [103]. CXCR2 ligands, namely, MIP2 (CXCL2) and KC (CXCL2) are the major ligands required for chemotactic signaling of neutrophils into brain abscesses as demonstrated by CXCR2 KO mice studies [92]. Impaired neutrophil influx into evolving brain abscesses in antibody-mediated neutrophil-depleted mice and CXCR2 KO mice resulted in exacerbated disease accompanied by elevated bacterial burdens compared to wild-type mice [92, 104]. In addition, chemokines such as CCL1, CCL2, CCL3, and CCL4 were also detected in evolving brain abscesses that most probably contribute to the influx of lymphocytes and monocytes and the establishment of the adaptive immune response [92].

Using a minocycline-resistant strain of *S. aureus* to dissect the antibiotic's bacteriostatic versus immune modulatory effects in a mouse experimental brain abscess model, minocycline was found to significantly reduce mortality rates within the first 24 hours following bacterial exposure [105]. This protection was associated with a transient decrease in the expression of several proinflammatory mediators, including IL-1*β* and CCL2. Minocycline was also capable of protecting the brain parenchyma from necrotic damage as evidenced by significantly smaller abscesses in minocycline-treated mice. In addition, minocycline exerted anti-inflammatory effects when administered as late as 3 days following *S. aureus* infection, which correlated with a significant decrease in brain abscess size. Finally, minocycline was capable of partially attenuating *S. aureus*-dependent microglial and astrocyte activation. This study suggests that minocycline may afford additional therapeutic benefits extending beyond its antimicrobial activity for the treatment of CNS infectious diseases typified by a pathogenic inflammatory component through its ability to balance beneficial versus detrimental inflammation.

## 3. Cytokines and Chemokines in Lyme Neuroborreliosis

Lyme borreliosis the most frequently reported vector-borne disease in the USA is caused by the spirochete *Borrelia burgdorferi (Bb)* [106]. It is transmitted through *Ixodes* ticks and is also prevalent in Europe and Asia [106, 107]. Lyme neuroborreliosis (LNB), the form of Lyme disease that affects the nervous system, manifests in about 15% of Lyme disease patients and affects both CNS and PNS [108–110]. Patients with CNS involvement complain of severe headaches, flu-like symptoms, fatigue, memory loss, learning disability, and/or depression. Infection of the PNS with *Bb* may result in facial nerve paralysis or palsy, pain in the back and limbs, and movement disorders.

Clinically, LNB may manifest as meningitis typically characterized by lymphocytic pleocytosis in the CSF, meningoradiculitis (a.k.a. Bannwarth's syndrome), cranial neuritis, encephalopathy, peripheral neuropathy, and, less commonly, encephalitis and encephalomyelitis. Radiculitis, or inflammation in the dorsal roots, is the most common manifestation of untreated Lyme borreliosis in humans [110]. LNB patients may also experience a wide array of neurological symptoms as a result of white matter inflammation that results in a subacute MS-like manifestation [111, 112]. Perivascular and vascular inflammatory processes may also occur in CNS LNB and several case reports of seizures or stroke have been attributed to neurologic Lyme disease [113–115].

Reports from human cases of LNB often include lymphocyte and plasma cell infiltration in the meninges and perivascularly in the nerve roots, dorsal root ganglia (DRG), and demyelination in the brain and spinal cord [113, 116–120]. Typically, PNS-Lyme disease is associated with transverse myelitis and patchy multifocal axonal loss with epineural perivascular inflammatory infiltrates or perineuritis [121–125]. The primary findings of axonal degeneration and regeneration and multifocal nerve lesions showing perivascular inflammatory cellular infiltrates have been documented in almost all patients with Lyme-associated peripheral neuropathy [123–127]. The results of these studies suggest that immune mediated neuronal and glial cell damage could be involved in the neuropathogenesis of LNB.

It is suggested that adherence of the spirochete to the endothelium lining of blood vessel walls leads to the release of inflammatory mediators [128]. This could in turn alter the permeability of the BBB and ensue entry of *Bb* into the CNS [128, 129]. The perivascular mononuclear cell infiltrates observed in the cerebral cortex during *Bb* infection consist predominantly of T-helper cells [130] and are associated with a focal increase in microglial cells and infiltration of lymphocytes and plasma cells in the leptomeninges [131].

Elevated levels of the proinflammatory cytokines IL-6, IL-8, IL-12, IL-18, and interferon *γ* [71–74] and the chemokines interferon-inducible T-cell chemoattractant (I-TAC), CCL2, CXCL-11, and CXCL13 [132–139] have been reported in the CSF of patients with LNB. The amount of IL-6 in human serum and CSF has been shown to correlate with disease activity in neurologic Lyme disease [132]. The chemokine CXCL13, which is known to attract B-lymphocytes, is also elevated in other instances of neuroinflammation [140]. CXCL13 expression in the CSF precedes the intrathecal production of *Bb*-specific antibodies [141] and may account for the high proportion of B-lymphocytes and plasma cells in the CSF of LNB patients, suggesting a role for the humoral immune response in Lyme neuroborreliosis [142].

Increased production of the neuromodulator quinolinic acid, an excitotoxin and N-methyl-D aspartate (NMDA) agonist, has been demonstrated in the CSF of patients with neurologic Lyme disease [143]. As the NMDA receptor mediates synaptic function and is involved in learning, memory, and synaptic plasticity [144], its dysregulation mediated by *Bb*-induced inflammation may contribute to the neurologic and cognitive deficits seen in many Lyme disease patients by mechanisms such as glutamate-mediated excitotoxicity [145].

The rhesus macaque is the preferred animal model for studying neurologic Lyme disease, as it exhibits most of the signs of Lyme disease seen in humans, both in the PNS and CNS [146, 147]. Using the monkey model, investigators have been able to examine the effect of *Bb* infection on neural tissue and its relationship to the adaptive and acquired immune responses. Since *Bb* itself does not produce any known endotoxin [148], damage to neural cells may occur in part due to bacterial lipoproteins that are present on the spirochetal surface or are released or shed by live or dead organisms. Lipidated outer-surface protein A (L-OspA), a prototype *Bb* lipoprotein, has been shown to induce IL-6, cell proliferation, and concomitant apoptosis in rhesus astrocytes *in vitro* [149, 150]. *Bb*-infected rhesus monkeys also showed astrogliosis in the frontal cortex [149]. In the presence of *Bb*, primary cultures of astrocytes or microglia have been shown to produce IL-6, IL-8, and the macrophage inflammatory proteins CCL3 and CCL4 [151]. Human neurons cocultured with *Bb* and rhesus microglia undergo apoptosis in the presence of proinflammatory mediators chiefly produced by the microglia [152].

In an *ex vivo* stimulation of monkey brain frontal cortex tissue explants with live *Bb*, IL-6, IL-8, IL-1*β*, and CXCL13 were visualized in glial cells, with concomitant oligodendrocyte and neuronal apoptosis [153], suggesting that the glial inflammatory response to *Bb* could contribute to cell death. In addition, microarray analyses of tissue RNA revealed altered transcription of multiple genes that regulate the immune response as well as apoptosis [153]. When live *Bb* was inoculated into the CNS of rhesus macaques via the cisterna magna [154], within one week after inoculation there was a monocytic and lymphocytic pleocytosis and increased expression of IL-6, IL-8, CCL2, and CXCL13 in the CSF. Histopathological changes consistent with acute neurologic Lyme disease, showing leptomeningitis and radiculitis, as well as satellite glial cell and neuronal apoptosis in the DRG were also observed. IL-6 was produced by both astrocytes and neurons of spinal cord tissue and by neurons in the DRG. The chemokines CXCL13 and CCL2 were detected in microglia of the spinal cord. CCL2 was also detected in endothelial cells in the periventricular area of the brain. Other investigators have also confirmed the production of IL-6 and CXCL13 in *B. burgdorferi*-infected rhesus and human tissues [155–157].

Patients with chronic and recurrent neurologic Lyme disease who have persistent symptoms even after treatment are plagued primarily by pain, fatigue, and cognitive dysfunction. Elevated levels of IL-6 can cause symptoms of fatigue and malaise, common to many infectious and neurodegenerative diseases. IL-6 is pyrogenic, promotes B cell differentiation, stimulates the synthesis of acute phase reactants, and can also contribute to pain by increasing the sensitivity of nerve endings [158]. It is possible that IL-6 mediates the pain response in the sensory neurons of the DRG in LNB as well, since it was seen in DRG neurons of infected rhesus macaques [155, 158]. 

In a recent study, live *Bb* was shown to elicit the production of cytokines and chemokines, particularly IL-6, IL-8, and CCL2, as well as to induce apoptosis in human oligodendrocytes cultured *in vitro* [159], by activating the enzyme caspase-3 [160]. Oligodendrocytes could therefore contribute to the elevated levels of cytokines and chemokines detected in the CSF of patients with LNB. Importantly, in the presence of the anti-inflammatory drug dexamethasone, a reduction in the amount of proinflammatory mediators, and a significant reduction in the *Bb*-induced oligodendrocyte apoptosis was observed [159]. This outcome is a strong indication that inflammation plays a role in mediating oligodendrocyte apoptosis, which could be mediated in part by the direct action of the spirochetes on oligodendrocytes or via inflammation mediated by *Bb* in oligodendrocytes.

Oligodendrocytes in brain tissue are especially vulnerable to demyelination as they are located immediately adjacent to the subarachnoid space, in the region known as the subpial space [161]. Oligodendrocytes are known to express receptors for various cytokines and chemokines [162]. Since inflammatory lesions are commonly found in the meninges in LNB, the myelitis that is seen in LNB may be in part due to oligodendrocyte dysfunction. These cells could be damaged by the inflammatory process initiated by the oligodendrocytes themselves, with participation of other glial cells, in addition to inflammatory mediators produced by the perivascular cellular infiltrates that are often present in CNS infection. As oligodendrocytes are vital for the survival and optimal function of neurons [162], oligodendrocyte damage could contribute to neuronal dysfunction and death and result in the impairment of CNS functions seen in patients with LNB. Caspase-mediated oligodendrocyte cell death has also been documented in inflammatory demyelinating diseases such as MS [163]. Cytokines and chemokines play a central role in inflammation, demyelination, and neurodegeneration in the CNS during inflammatory neurodegenerative diseases such as MS, which shows similar clinical signs as those shown by LNB [164].

The chemokine CCL2 reported in Lyme neuroborreliosis is of particular importance in mediating inflammation in neurodegenerative diseases [165, 166]. It is an important mediator in many neuroinflammatory and neurodegenerative brain diseases characterized by neuronal degeneration [167]. CCL2 has been found to be upregulated in actively demyelinating MS plaques [168], and its expression is increased in experimental autoimmune encephalomyelitis [169]. CCL2 modulates microglial activation and proliferation, thereby contributing to the inflammatory response mounted in the CNS [170]. Importantly, CCL2 levels are elevated in the CSF of patients with LNB [136], and high levels of CCL2 have been found in the CSF of rhesus monkeys infected intrathecally with *Bb* [154]. CCL2 is known to play a role in mediating nerve damage and demyelination of axons by causing an influx of monocytes and T cells in Wallerian degeneration [171] that may also possibly contribute to the axonal damage that affects patients with LNB of the PNS [172].

The cytokine IL-6 that has been reported in studies of LNB pathogenesis is known to be both helpful and harmful in the CNS [15, 173–176]. Dysregulated expression of IL-6 has been documented in several neurological disorders such as MS, acute transverse myelitis, AD, schizophrenia, epileptic seizures, and PD [177]. In addition, IL-6 has been shown to be involved in multiple physiological CNS processes such as neuron homeostasis, astrogliogenesis, and neuronal differentiation [178]. IL-6 is known to promote oligodendrocyte and neuronal survival in the presence of glutamate-mediated excitotoxicity in hippocampal slices [179] and promotes survival of oligodendrocytes *in vitro* [180]. It is possible that IL-6 could mediate both neuroprotection as well as neurodegeneration in inflammatory neurodegenerative diseases including LNB.

The chemokine IL-8, also seen to be elevated in the CSF of LNB patients [132, 179] and in rhesus microglia, astrocytes, and endothelial cells exposed to *Bb* [151–154], is associated with BBB dysfunction and plays a central role in recruitment of neutrophils and T cells into the CNS during bacterial meningitis [181–183]. IL-8 is known to induce the expression of proinflammatory proteases, the matrix metalloproteinases MMP-2 and MMP-9, and proapoptotic protein Bim (Bcl-2-interacting mediator of cell death) and cell death, in cultured neurons in 24 hours [184]. There are reports indicating the presence of a cytolytic phenotype of IFN-*γ*-producing cells from patients with LNB [185, 186], suggesting the possible involvement of cytotoxic cells in mediating the demyelination and axonal degeneration seen in LNB [113, 116, 121].

*B. burgdorferi* has also been shown to induce the late production of significant quantities of the anti-inflammatory cytokine IL-10 in murine microglia and astrocytes [187]. The delayed production of IL-10 suggests that a possible negative feedback loop to limit potentially damaging inflammation within the brain parenchyma during persistent infections may be operating in parallel with the harmful effects of proinflammatory mediators. The treatment of primary rhesus macaque microglia with the tetracycline analogs doxycycline and minocycline resulted in attenuated microglial proinflammatory mediator responses to *Bb* [188]. Doxycycline is used for the treatment of Lyme disease patients and has been shown to improve adverse clinical symptoms at a time when viable spirochetes can no longer be easily detected. Therefore, the dampening of microglial proinflammatory cascades to limit unchecked neuroinflammation and subsequent neuronal damage may be beneficial to LNB patients.

## 4. Cytokines and Chemokines in Human Immunodeficiency Virus Encephalitis

Infection with the human immunodeficiency virus-1 (HIV-1) and acquired immunodeficiency syndrome (AIDS) are a persistent health problem worldwide. HIV-1 seems to enter the brain very soon after peripheral infection and can induce severe and debilitating neurological problems that include behavioral abnormalities, motor dysfunction, and dementia. The neurological manifestations directly related to HIV are acute viral meningitis, chronic meningitis, HIV-associated dementia (HAD), vacuolar myelopathy, and involvement of the peripheral nervous system [189]. Infected peripheral immune-competent cells, in particular macrophages, appear to infiltrate the CNS and provoke a neuropathological response involving all cell types in the brain. HIV-1 encephalitis (HIVE), a common pathological manifestation of HAD, includes infiltration of macrophages into the brain where they become productively infected with the virus. This is accompanied by considerable cytokine and chemokine dysregulation in the brain that often culminates into the unique pathological features that characterize this syndrome. Once in the brain, HIV-1-infected blood-borne macrophages secrete proinflammatory cytokines such as TNF, IL-1*β*, and viral proteins such as HIV-1gp120 and Tat, which can affect neuronal function [190]. In the CNS, HIV-1 also incites activation of chemokine receptors, inflammatory mediators, extracellular matrix-degrading enzymes, and glutamate receptor-mediated excitotoxicity, all of which can initiate numerous downstream signaling pathways and disturb neuronal and glial function.

Lentiviruses are thought to enter the brain within circulating infected monocytes during immune surveillance. Numerous studies have been undertaken to determine the reasons underlying increased monocyte migration into the brain following lentiviral infection. HIV-infected leukocytes are primed for adhesion [191], having already shed L-selectin and increased expression of CD11b/CD18 compared with monocytes from healthy controls [192]. Therefore, it is possible that even marginal increases in the levels of chemokines expressed within the parenchyma would lead to increased migration of monocytes. Recent studies have shown that glial cells are stimulated to produce chemokines in response to inflammatory cytokines [193, 194] that are known to be secreted by simian immune-deficiency-virus- (SIV-)infected macrophages [195].

### 4.1. Astrocytes and Signaling in HIV Encephalitis

Astrocytes act to repel circulating immune cells through secretion of eotaxin [196], reinforcing the brain's immune-privileged status in conjunction with the selective physical properties of the BBB. Under normal conditions the brain allows only limited access by immune cells. Early in HIV infection the virus enters the brain through normal trafficking. This leads to a transient increase in BBB permeability and a localized immune response. As the disease progresses to encephalitis, the immune response is dramatically increased, marked by a loss of tight junction integrity, gliosis, and formation of multinucleated giant cells in the parenchyma.

Astrocytes are the primary cell type found in glia scar formation [197, 198], and secrete cytokines and chemokines to elicit increased trafficking of leukocytes into the brain [193, 199, 200]. Astrocytes may also provide a role for the resolution of inflammation by reducing the secretion of proinflammatory cytokines, and increasing anti-inflammatory processes [198, 201, 202]. Decreased BBB integrity early in SIV/HIV infection allows latently infected monocytes to enter the brain [203]. Circulating virus could induce brain microvessel endothelial cells (BMEC) to express CD106 diffusely [204, 205] leading to increased monocyte migration into brain, where they become productively infected. Astrocytes respond to these macrophages resulting in a wide-range of cellular changes referred to as astrogliosis.

### 4.2. Astrogliosis

On activation astrocytes undergo a morphological change, most notably an increase in ramification concomitant with upregulation of GFAP and thickened processes. Some astrocytes in the proximity of SIV lesions express peripherin, an alternative type III intermediate filament not normally expressed in brain [206]. Immunologically, astrocytes respond to HIV/SIV infection through increased production of inflammatory cytokines. As outlined above, the predominant inflammatory cell type in HIVE/SIVE is the monocyte-derived macrophage. The chemokines upregulated by astrocytes in HIVE/SIVE are largely specific to monocyte/macrophages [193, 207]. This suggests the possibility of a positive feedback system being initiated; a productively infected macrophage induces nearby astrocytes to up-regulate secretion of macrophage-specific chemokines, leading to lesion formation. The cytokine response of astrocytes includes a cornucopia of molecules including a variety of chemokines. It is intriguing that astrocytes will secrete a different “profile” of cytokines and chemokines in response to different classes of stimuli [208]. Below we discuss key cytokines and chemokines that are thought to play a role in SIVE/HIVE.

### 4.3. Microglia Activation and Cytokine Secretion

Microglia serve as a “first responder” to neuroinvasion by pathogens. An actin binding protein, AIF-1, is considered to be a microglial-specific marker within normal brain [209–211]. As such, AIF-1 is ideally suited to examining morphological changes in microglia. Ramified microglia sample their environment using long processes. These processes retract on activation, allowing the microglia to migrate to the source of infection [212]. Cultured microglia also have a ramified morphology until activated by, for example, SIV-infected macrophages [213].

Surprisingly, the presence of macrophages is more important to the microglial response rather than whether they are infected with virus or not. IL-6 and IL-8 are both induced to be secreted by microglia when coincubated with macrophages, highlighting their role as “first responders” to infiltrating innate immune cells such as monocyte/macrophages. 

### 4.4. Expression and Secretion of Selected Cytokines

Productively infected macrophages in the encephalitic brain express TNF [195]. TNF-*α* receptors are present in the nonencephalitic brain [214], such that normal brains are primed to respond quickly to low levels of TNF. TNF induces increased chemokine production and secretion by astrocytes [215], and these chemokines induce monocyte migration preferentially over lymphocytes [193].

Vascular endothelial growth factor (VEGF) promotes proliferation of BMEC, resulting in reorganization of the cytoskeleton and tight junction proteins. This induces a decrease in BBB integrity, creating a permissive environment for monocyte migration, and also bidirectional leakage of proteins across the BBB. A possible mechanism for the VEGF pathway could be as follows: tat binds to the VEGF receptor [216], followed by the binding of the VEGF receptor to focal adhesion kinase [217], increases of which have been implicated in BBB disruption [218].

Other proinflammatory cytokines, including IFN-*γ* and IL-6, are upregulated in the encephalitic brain, with far-reaching effects in neuroinflammatory events [219]. The complement pathway is also known to be induced through IFN-*γ* and IL-6 signaling, resulting in propagation of inflammation in the area surrounding lesions. There are well-characterized neurotoxicity manifestations associated with HIV infection [220], including increased secretion of the neurotoxic IL-6 by glia in response to gp120 [221]. Therefore, rapid secretion of high levels of IL-6 by microglia would be anticipated to be a detrimental effect of SIV-infected macrophage infiltration into the brain [222].

### 4.5. Expression and Secretion of Selected Chemokines

Levels of the chemokine IL-8 has been recently demonstrated to be elevated in microglia in HIVE brain tissue [223], possibly in response to gp120 [224]. IL-8 have been shown to have neurotoxic effects and thus, plays a role in cognitive dysfunction associated with HIV [225]. Increased IL-8 expression observed in glial nodules may be largely due to a factor secreted by HIV-infected macrophages [213].

An early study of chemokine expression in brains of macaques infected with SIV showed increased CCL3, CCL5, CCL7, and CXCL10 [207], although no increase in CCL2, CCL8 (MCP-2), or CXCL8 was observed in this study. Other later studies have produced conflicting results. Penton-Rol used dexamethasone to stimulate cells to have increased CCL2 receptors before infecting with HIV 89.6 [226]. The Clements group at Johns Hopkins has shown increased CCL2 mRNA in brain extracts using a highly accelerated encephalitis model [227], although mRNA does not always equate with secreted protein. Additionally, the Berman group at Einstein College of Medicine has shown numerous effects of CCL2 on HIV-infected macrophages [200, 228]. CCL2 was among several chemokines in CSF that were not upregulated in one study using humans infected with HIV [229], although IP-10 was upregulated. In contrast, CCL2 was increased in pigtail macaques that develop encephalitis [230]. The precise cell types producing these chemokines were not identified in these studies. CCL2 mRNA was upregulated in cultured astrocytes, but remained at low levels compared to CCL7, suggesting a role for CCL7 in HIV-related encephalitis [193].

Even under noninflamed conditions, CCL7 is expressed in the brain [193, 207], which could contribute to basal levels of monocyte migration into the brain for “routine surveillance” [231]. That CCL7 is upregulated by astrocytes in response to cytokines present in encephalitic brains gives a potential role for controlling monocyte migration during encephalitis as well [193, 207]. In more recent studies, stimulation of astrocytes with TNF induced an increase in secretion of numerous cytokines [215]. Analyses of gene arrays of astrocytes treated with TNF showed that the only cytokine upregulated was CCL7. It is also possible that astrocytes provide a role for the resolution of inflammation through reduction in secretion of proinflammatory cytokines and increasing anti-inflammatory processes [198, 201, 202]. In the above study, polygonal astrocytes stimulated with TNF expressed higher levels of cytokines including VEGF than TNF-stimulated stellated astrocytes at the time points examined. Therefore, the order of stimulation of astrocytes is important in the subsequent secretion of cytokines [215]. 

## 5. Cytokines and Chemokines in Neuropathic Pain

Recent evidence suggests a strong correlation between inflammation following nerve damage and neuropathic pain [10, 23, 232]. Neuropathic pain is a complex syndrome resulting from many forms of peripheral nerve damage, for example, traumatic nerve injury, diabetes, infection, or drug-induced neuropathy, and immune and metabolic diseases [233]. Chronic pain can occur with peripheral nerve trauma and/or inflammation, autoimmune neuropathies and vasculitic neuropathies, or infection. Individuals who suffer from chronic pain experience prolonged pain at sites that may have been previously injured, yet are otherwise currently healthy. Chronic pain is associated with changes in neuroplasticity or changes in neural pathways, and synapses due to an erroneous reorganization of the nervous system, both peripherally and centrally. During the period of tissue damage, noxious stimuli and inflammation cause an elevation of nociceptive input from the periphery to the central nervous system. Prolonged nociception from the periphery elicits a neuroplastic response at the cortical level to change its somatotopic organization for the painful site, inducing central sensitization [234].

Peripheral nerves are the origin of almost all forms of neuropathic pain. Pain-responsive peripheral nerves reveal a remarkable degree of plasticity in both sensory neurons and spinal cord [10]. Immune processes can be directed against peripheral nerves, DRG, and dorsal roots resulting in pathological pain. 

Immune activation near peripheral nerves may create increases in peripheral nerve excitability. Infectious agents as well as proinflammatory mediators produced by activated microglia can cause alterations in the blood-nerve barrier (BNB) as a result of chemoattractant molecules released at the site of the damaged peripheral nerve, which in turn recruit neutrophils and macrophages from the circulation into the nerve. Proinflammatory cytokines participate in this immune activation and orchestrate the early immune response. However, these inflammatory mediators can directly increase nerve excitability, damage myelin, and alter the permeability of the BNB leading to edema and further infiltration of immune cells. Schwann cells that ensheath peripheral nerves are macrophage-like and can present nonself-substances to T lymphocytes to further activate the immune cells. Schwann cells also participate in the removal of damaged myelin and cellular debris. Importantly, Schwann cells rapidly release the chemo-attractant CCL2 upon nerve damage that in turn recruits monocytes and T cells to the site of the nerve degeneration [10]. Proinflammatory cytokines have been repeatedly implicated in demyelination and degeneration of peripheral nerves, increases in sensory afferent excitability, and induction of neuropathic pain [23].

Inflammatory mediators elicited in the cells of the DRG and those produced by infiltrating immune cells and spinal microglial activation are key elements that mediate the signal transduction of the pain response [235]. Macrophages, lymphocytes, and satellite glial cells in the DRG and in the dorsal horn of the spinal cord participate in neuroimmune activation of glial cells, promoting the development of neuropathic pain. Since some chemokine receptors such as CCR2, CCR5, CXCR4, and CX3CR1 are located in primary afferent neurons or secondary neurons of the spinal dorsal horn [236], their chemokine ligands can potentially alter pain transmission. Peripheral administration of the chemokines CCL2, CCL3, CCL5, and CXCL12 has been shown to produce pain behaviors that are elicited by the activation of chemokine receptors in the DRG [236].

Importantly, CCL2 participates in pain regulation by directly interacting with sensory neurons and indirectly via peripheral leukocyte activation in the PNS [236, 237]. CCL2 has been shown to be elevated in primary sensory neurons after nerve injury, and CCR2 expression has been observed in both DRG neurons and activated Schwann cells in injured peripheral nerves [237]. Moreover, neuropathic pain induced by nerve injury is not elicited in CCR2 gene-deficient mice [236]. The addition of CCL2 to cultured DRG neurons triggered the release of calcitonin gene-related peptide (CGRP), a nociceptor neurotransmitter, from these cells, presumably as a result of increased neuronal excitation [238].

CCL3 has been found to be upregulated in activated Schwann cells and in infiltrating macrophages close to injured nerves. This chemokine participates in the development of neuropathic pain through its receptors CCR1 and CCR5, which are located in Schwann cells and macrophages [239]. Interestingly, there is an increase of fractalkine (CX3CL1), which is known to be both a pro- and anti-inflammatory molecule in injured nerves, and localization of its receptor CX3CR1 in recruited macrophages and DRG neurons. The activation of fractalkine-CX3CR1 has been shown to attenuate peripheral nerve injury-induced neuropathic pain [240].

The crosstalk between glial cells and neurons is important in the development of neuropathic pain [234]. Proinflammatory cytokines such as IL-1*β*, IL-6, and TNF produced by glial cells and neurons accelerate central pain sensitization, and inhibition of these cytokines in the CNS and PNS effectively reduces neuropathic pain [241]. Brain-derived neurotrophic factor (BDNF) derived from activated microglia potentiates the excitability of spinal neurons [242]. Microglial IL-18, a member of the IL-1 family, also plays a pivotal role in neuropathic pain [243]. IL-1*β* produced by macrophages and Schwann cells in injured nerves directly sensitizes nociceptors in primary afferent neurons [244]. IL-1 induces the release of substance P from DRG neurons [245] and neuropathic pain is reduced in IL-6 KO mice [246]. IL-6 can also contribute to pain by increasing the sensitivity of nerve endings [246]. IL-6 can enhance neuropathic pain in the dorsal horn by activating STAT3 signaling in glial cells after peripheral nerve injury. The STAT-3 pathway is a key mediator of signal transduction in neuropathic pain [247].

A recent study evaluated the role of axonal transport in neuroimmune communication following peripheral nerve injury, linking focal changes in Schwann cell activation and release of the proinflammatory cytokine TNF with subsequent activation and sensitization of ascending sensory neurons and glia that culminate in the neuropathic pain state. New data demonstrate that axonally transported (biotinylated) TNF-*α* activates and localizes with dorsal horn astrocytes within 96 hours after injection into the sciatic nerve and that glial GFAP-activation in these glial cells is diminished in TNF-*α*-receptor 1 KO mice [248]. 

IL-17 is an important regulator of immune responses and is involved in inducing and mediating proinflammatory reactions in a wide range of inflammatory and autoimmune diseases of the nervous system. Using IL-17 KO mice, it has been demonstrated that IL-17 contributes to neuroinflammatory responses and pain hypersensitivity following neuropathic injury [249]. Compared to wild-type, IL-17 KO mice displayed significantly decreased mechanical pain hypersensitivity as well as decreased infiltration of T cells and macrophages to the injured sciatic nerves, and the L3-L5 DRG, and decreased activation of microglia and astrocytes in the L3-L5 dorsal and ventral horns of the spinal cord. This work shows that IL-17 contributes to neuroinflammation and neuropathic pain following peripheral nerve injury and identifies IL-17 as a potential therapeutic target for treating neuropathic pain.

Recent studies have suggested that the C-C chemokine receptor (CCR)5 interacts with *μ*-opioid receptor and modifies a nociceptive reaction [250]. A study that examined effects of CCR5 deficiency on pain responses by employing CCR5 KO mice found that pain responses of CCR5 KO mice to chemical or inflammatory stimuli were milder than those of CCR5 wild-type mice [251]. Though the roles of proinflammatory cytokines and chemokines in neuropathic pain have been identified [252], the precise relationship between the chemokine-cytokine network and neuropathic pain is not yet well understood. Further studies are needed to understand the neuropathic regulatory mechanisms underlying neuroinflammation after nerve injury.

## 6. Conclusion

Cytokines and chemokines play an important role in mediating neuroinflammation and neurodegeneration in various kinds of inflammatory neurodegenerative diseases including bacterial meningitis, brain abscesses, Lyme neuroborreliosis, and HIV encephalitis described above. Interestingly, recent evidence suggests that peripheral and central neuroinflammation associated with cytokine-chemokine networks following nerve damage also play a central role in the pathogenesis of neuropathic pain. Although a link has been established between cytokines, chemokines, and neurodegeneration, their signaling mechanisms are complex and appear to involve a balance between promoting cell survival, apoptosis, and proinflammatory responses.

Although inflammation may induce beneficial effects such as pathogen clearance and phagocytosis of debris and apoptotic cells besides tissue repair processes, uncontrolled inflammation can result in detrimental outcomes via the production of neurotoxic factors that exacerbate neurodegenerative pathology. The factors that may disrupt this normal equilibrium remain largely unknown. Several cytokines and chemokines and their receptors orchestrate this immune response. Further, anti-inflammatory responses are regulated by proteins that inhibit signal transduction pathways, such as suppressor of cytokine signaling proteins, transcriptional repressors, and anti-inflammatory molecules that help control excessive inflammation. Chemokine recruitment of inflammatory cells to the sites of injury is instrumental in driving a secondary damage cascade. 

Given the mounting evidence for their role in neurodegenerative disorders, cytokines and chemokines have received considerable attention as therapeutic targets. Considering that inflammation mediated by cytokines and chemokines is a common denominator in neurodegenerative diseases, targeting the correct timing of an immune response will be a pivotal factor in designing successful therapies. Further, it will be a challenge to design therapeutic agents that safely and effectively target only the detrimental mechanisms that contribute to disease pathogenesis, as cytokines and chemokines are vital for the normal functioning of the body. An understanding of the factors that dictate the switch from a protective to a deleterious inflammatory response will make possible interventions able to limit tissue damage. This type of intervention will also require a thorough understanding of the cell-subtype-specific action and cellular signaling pathways in CNS and PNS injury in order to design selective drug targets. Further investigations aimed at understanding chemokine-cytokine networks that are operative in the signal transduction of the pain response will prove beneficial in designing novel therapeutic strategies to alleviate neuropathic pain, a significant factor in neurodegenerative diseases.
